# Preliminary exploration of the value of pericoronary adipose tissue radiomics in identifying high-risk patients with chronic coronary syndrome: a retrospective Chinese cohort study

**DOI:** 10.3389/fmed.2026.1797905

**Published:** 2026-09-03

**Authors:** Zhang Ludan, Chen Chen, Zhang Yu, Hou Hui, Wang Zewen, Xia Xiaofeng, Luo Xiangwei, Zeng Yan, Chen Jianzhou

**Affiliations:** 1 Department of Radiology, 901st Hospital of the Joint Logistic Support Force of the People’s Liberation Army of China, China; 2 Department of Medical Imaging, Taizhou Fourth People’s Hospital, Taizhou, China; 3 Taizhou Teaching Hospital, Anhui University of Science and Technology, Taizhou, China; 4 Shanghai United Imaging Intelligence Co., Ltd., Shanghai, China

**Keywords:** chronic coronary syndrome, pericoronary adipose tissue, radiomics, risk stratification, tomography, x-Ray computed

## Abstract

**Background:**

Chronic coronary syndrome (CCS) requires accurate risk stratification for optimized management. Pericoronary adipose tissue (PCAT) inflammation is closely associated with plaque progression, and coronary computed tomography angiography (CCTA)-based radiomics may provide information beyond the traditional fat attenuation index (FAI).

**Objective:**

To assess the value of PCAT radiomics and its combination with one-stop CCTA parameters for identifying CCS patients who are classified as high-risk by the 2024 European Society of Cardiology (ESC) guideline-recommended risk factor-clinical (RF-CL) model.

**Methods:**

We retrospectively enrolled 175 CCS patients who underwent CCTA between January 2024 and January 2026. According to the 2024 ESC guideline-recommended RF-CL model, patients were classified as high risk (>15%, *n* = 77) or low risk (≤15%, *n* = 98). PCAT radiomics features were extracted from the three major coronary arteries, and FAI, computed tomography-derived fractional flow reserve (CT-FFR), coronary artery calcium score (CACS), and the Coronary Artery Disease Reporting and Data System (CAD-RADS) were analyzed.

**Results:**

Significant between-group differences were found in right coronary artery (RCA) FAI, left anterior descending (LAD) FAI, CACS, CAD-RADS, distal CT-FFR at the most stenotic plaque, and distal vessel CT-FFR, but not left circumflex (LCX) FAI. Distal vessel CT-FFR showed better discrimination than lesion-specific CT-FFR (area under the receiver operating characteristic curve [AUC] 0.782 vs. 0.698). The RCA PCAT radiomics model outperformed RCA FAI in the testing set (AUC 0.729 vs. 0.620). The integrated model achieved the best performance, with AUCs of 0.860 and 0.823 in the training and testing sets.

**Conclusion:**

PCAT radiomics, especially when integrated with one-stop CCTA parameters, improves the identification of CCS patients classified as high-risk by the RF-CL model. Prospective studies with hard cardiovascular outcomes are required to validate its prognostic value and to determine whether this imaging-based strategy can guide therapeutic decision-making.

## 1 Introduction

Chronic coronary syndrome (CCS) encompasses a spectrum of clinical presentations ranging from stable angina to ischemic cardiomyopathy. The cornerstone of its long-term management lies in the accurate assessment of a patient's risk for future major adverse cardiovascular events (MACE) to guide the intensity and strategy of individualized therapy (1–3). Traditional risk assessment models primarily rely on clinical symptoms, demographic characteristics, and conventional risk factors. In recent years, coronary computed tomography angiography (CCTA) has emerged as a first-line non-invasive tool for evaluating CCS. It not only diagnoses anatomical stenosis but also provides information on plaque characteristics, perivascular inflammation, and hemodynamic function (4). Among these, pericoronary adipose tissue (PCAT) has garnered significant attention due to its close anatomical relationship with the coronary vessel wall and its active paracrine function (5). Research indicates (6) that the inflammatory status of PCAT can be reflected on CCTA through changes in its attenuation value, quantified as the fat attenuation index (FAI). This metric is associated with coronary inflammation and plaque instability. Furthermore, CT-derived fractional flow reserve (CT-FFR) can non-invasively assess the hemodynamic significance of a stenosis, while the coronary artery calcium score (CACS) and the Coronary Artery Disease Reporting and Data System (CAD-RADS) grade quantify calcific burden and stenosis severity, respectively (7–9). To integrate these multi-dimensional data, recent clinical guidelines recommend using combined clinical risk models (e.g., the RF-CL model) for more precise risk stratification. Concurrently, radiomics, an emerging image analysis methodology, enables the high-throughput extraction of a vast number of quantitative texture and morphological features from medical images that are imperceptible to the human eye, thereby offering deeper insights into tissue heterogeneity and pathophysiological states. Preliminary studies suggest that CCTA-based PCAT radiomic features hold substantial promise for discriminating plaque stability and predicting cardiovascular events (10).

Although FAI, as an imaging biomarker of PCAT inflammation, has been extensively studied, its clinical utility remains debated. In particular, findings regarding its consistency across different coronary territories and its predictive capacity for long-term outcomes are inconsistent across studies. For instance, some research has identified uncertainty in the evaluative value of FAI around the left circumflex artery (11), while long-term follow-up studies have even suggested that its predictive power for MACE may be limited (12). CT-FFR primarily reflects the immediate hemodynamic impact of a specific stenosis, and CACS mainly reflects long-term calcific burden; neither directly quantifies the active inflammatory process surrounding the vessel wall. Although contemporary comprehensive risk models like RF-CL have significantly improved discrimination, their core still relies on clinical parameters, with insufficient integration of novel biological information provided by imaging (12). While radiomics can provide deeper information beyond traditional parameters like FAI, its application in CCS risk stratification remains in its infancy. Most studies have limited sample sizes and lack systematic comparison and combined validation with established metrics such as FAI, CT-FFR, CACS, and CAD-RADS within a unified framework. More importantly, it is currently unclear how a PCAT radiomics-based model performs in identifying high-risk CCS patients as determined by modern comprehensive risk models (e.g., RF-CL), and whether its combination with the aforementioned multi-parametric, one-stop CCTA parameters can construct a superior risk stratification tool (13). Existing research predominantly focuses on predicting long-term hard endpoints. There remains a paucity of in-depth exploration into utilizing these advanced imaging tools to optimize immediate clinical risk stratification for the precise identification of high-risk individuals currently requiring intensive intervention (14).

Therefore, this study aims to systematically investigate the value of CCTA-based PCAT radiomic features, both alone and in combination with various imaging indices including FAI, CT-FFR, CACS, and CAD-RADS grading, in identifying CCS patients who are classified as high-risk by the RF-CL model. By constructing and comparing the diagnostic performance of different models, this research expects to provide evidence for establishing a more precise, novel multi-parametric imaging-based CCS risk stratification strategy, thereby advancing individualized clinical management.

## 2 Materials and methods

### 2.1 Study population

This was a multicenter, retrospective, observational study designed to evaluate the value of CCTA-based PCAT radiomic features in identifying high-risk CCS patients. We consecutively enrolled patients hospitalized in the Department of Cardiology at the 901 Hospital of the Joint Logistics Support Force of the People's Liberation Army (PLA), Hefei, China, and Taizhou Fourth People's Hospital, Taizhou, Jiangsu, China, between January 2024 and January 2026. Inclusion criteria were a clinical diagnosis of CCS and completion of a CCTA examination. The study protocol was approved by the Ethics Review Committee of the 901 Hospital of the Joint Logistics Support Force of the PLA (Approval No.: 202508001). Informed consent was waived due to the retrospective design and stringent protection of patient privacy.

Sample size calculation was based on the requirements for predictive model research and referenced recent similar radiomics studies (2). Estimation was performed using PASS 15.0 software. The significance level (*α*) was set at 0.01 (two-sided, Bonferroni-adjusted for the five main imaging predictors assessed) and the statistical power (1−*β*) at 0.90. Based on pilot data, the anticipated AUC for the combined model was 0.85, compared to a null AUC of 0.70 for a traditional imaging model. The calculated minimum sample size was 160 patients. Accounting for an estimated attrition rate of approximately 10% during data screening, a minimum of 175 patients were planned for final inclusion to ensure sufficient statistical power for model construction and validation. The final test set AUC of 0.823 had a 95% confidence interval of 0.682–0.964, which, while including the null value 0.70, is compatible with clinically meaningful discrimination and is explicitly noted as a limitation of the current sample size. Considering an estimated attrition rate of about 15% during data screening, a minimum of 175 patients were planned for final inclusion to ensure sufficient statistical power for model construction and validation.

Between January 2024 and January 2026, a total of 287 patients with a clinical diagnosis of CCS who underwent CCTA at the two participating centers were initially screened. According to the exclusion criteria, 38 patients were excluded because of a prior history of coronary artery bypass grafting or percutaneous coronary intervention; 22 patients were excluded because CCTA image quality was insufficient for post-processing analysis (severe motion artifacts or excessive noise); 18 patients were excluded because of incomplete clinical data or laboratory results; 24 patients were excluded because of comorbid severe arrhythmia (e.g., persistent atrial fibrillation), left ventricular ejection fraction <40%, congenital heart disease, anomalous coronary artery origin or course, or myocardial bridge causing >50% luminal compression; and 10 patients were excluded because CCTA demonstrated total occlusion of any major coronary artery (left main, left anterior descending, left circumflex, or right coronary artery). Ultimately, 175 patients met all criteria and were included in the final analysis. According to the RF-CL model recommended by the 2024 ESC guidelines for CCS management, 98 patients were classified into the low-risk group (≤15%) and 77 into the high-risk group (>15%). Importantly, because the RF-CL score was derived exclusively from clinical variables and did not incorporate any CCTA-derived parameters, the significant differences in imaging metrics (e.g., CACS, CT-FFR, FAI, radiomics) between risk groups represent genuine pathophysiological associations rather than circular reasoning. Baseline characteristics including age, sex, and cardiovascular risk factors were compared between the two groups.

Inclusion Criteria:

(1) Age ≥18 years;
(2) Clinical diagnosis of CCS according to relevant international guideline criteria;
(3) Underwent complete CCTA scanning, including non-contrast and contrast-enhanced phases, during hospitalization, with complete and retrievable imaging data.

Exclusion Criteria:

(1) Prior history of cardiac surgery (e.g., coronary artery bypass grafting);
(2) Prior history of revascularization procedures such as percutaneous coronary intervention;
(3) CCTA images with severe artifacts or noise, unanimously assessed by two or more physicians as unsuitable for precise coronary and perivascular tissue analysis;
(4) Missing key clinical baseline data (e.g., lipid profiles, blood glucose) or imaging data;
(5) Comorbid severe arrhythmia (e.g., atrial fibrillation) or severe cardiac dysfunction (New York Heart Association class III-IV) potentially affecting image quality and analysis;
(6) Presence of congenital heart disease, anomalous coronary artery origin/course, or hemodynamically significant myocardial bridge;
(7) CCTA showing total occlusive disease in any major epicardial coronary artery.

### 2.2 Risk stratification and clinical data collection

The RF-CL model recommended by the 2024 ESC guidelines was used to define the reference standard for high-risk status. The RF-CL score was calculated for each patient based exclusively on clinical variables: age, sex, symptom characteristics (typical angina, atypical angina, non-anginal chest pain), and cardiovascular risk factors (current smoking, diagnosed hypertension, diabetes mellitus, total cholesterol, and high-density lipoprotein cholesterol). No CCTA-derived features—including CACS, CT-FFR, FAI, CAD-RADS, or any radiomic variable—were incorporated into the RF-CL classification. Thus, the imaging predictors in our combined model are entirely independent of the endpoint definition, ensuring that the analysis assesses the ability of imaging to identify patients already categorized as high-risk by a purely clinical model. Patients with an estimated 10-year risk of cardiovascular death or non-fatal myocardial infarction >15% were assigned to the high-risk group; those with a risk ≤15% were assigned to the low-risk group. Clinical and laboratory data, including cardiovascular risk factors, medication use, and laboratory results, were retrieved from the electronic medical record system.

### 2.3 Image acquisition protocol

All CCTA examinations analyzed in this retrospective study were retrieved from the institutional picture archiving and communication system (PACS). These examinations had been performed for clinical indications before study enrollment using one of two high-end CT scanners: a 160-detector-row, 320-slice helical CT scanner (uCT 860; United Imaging Healthcare, Shanghai, China) or a dual-source CT (Siemens SOMATOM Force). Clinical and laboratory data were retrieved from the electronic medical record system, as described in the Study Population section. For patients with a resting heart rate >65 beats per minute, oral beta-blockers (metoprolol tartrate) were administered as needed to lower and stabilize the heart rate. The scan range extended from approximately 1 cm below the tracheal bifurcation to below the cardiac diaphragm. The scanning protocol included a non-contrast coronary calcium scan followed by contrast-enhanced coronary CTA.

For the contrast-enhanced scan, a bolus-tracking trigger technique was employed. A region of interest was placed in the pulmonary trunk or descending aorta to monitor the dynamic change in CT attenuation. When the attenuation reached a pre-set trigger threshold (120–150 Hounsfield Units), the coronary phase scan was automatically initiated. Using a dual-syringe power injector, iohexol contrast medium (350 mg I/mL) was administered via an antecubital vein at a flow rate of 5.0 mL/s, with a dose calculated at 1.0 mL/kg body weight, followed by a 50 mL saline flush at the same rate.

Scan parameters were as follows: tube voltage was fixed at 120 kV. Automatic tube current modulation was applied to optimize radiation dose. Gantry rotation times were 0.33 s/rotation (uCT860) and 0.25 s/rotation (SOMATOM Force). Raw data were reconstructed with a slice thickness of 0.75 mm and a slice increment of 0.75 mm using a dedicated cardiac convolution kernel to obtain axial images for subsequent analysis.

### 2.4 Study procedures

(1) Pericoronary Fat Attenuation Index Measurement (15): CCTA source image data were imported into the dedicated fat analysis module of the United Imaging Intelligent Research Platform. Following established methodology, the software automatically delineated a tubular volume of interest extending radially 4 mm outward from the outer vessel wall of the left anterior descending (LAD), left circumflex (LCX), and right coronary artery (RCA) main stems. Specific analysis segments were defined as: LAD and LCX from the ostium to 40 mm distally; RCA from 10 mm distal to the ostium to 50 mm distally. Attenuation values of adipose tissue within this region were constrained between −190 and −30 Hounsfield Units to exclude non-adipose tissue. The software automatically calculated the mean FAI for each vessel, recorded as LAD-FAI, LCX-FAI, and RCA-FAI.

(2) Non-invasive Fractional Flow Reserve Calculation (16): In accordance with recommendations from the Chinese Expert Consensus on the Clinical Pathway for CT-FFR Application, CT-FFR analysis was performed using integrated workstation software (RuiXin-FFR 2.0.3). CT-FFR analysis was performed by an experienced cardiac imager who was blinded to the RF-CL classification and all clinical risk data. For each of the three major epicardial coronary arteries (LAD, LCX, RCA), two CT-FFR values were recorded: (1) plaque-distal CT-FFR, measured 1 cm distal to the most severe stenosis; (2) distal vessel CT-FFR, measured at a distal vessel diameter of approximately 2 mm. In vessels with no obstructive stenosis on CCTA, the distal vessel CT-FFR was measured at the same standardized distal location (approximately 2 mm diameter). The patient-level distal vessel CT-FFR was defined as the lowest value among the three vessels. This metric was chosen because it integrates the cumulative hemodynamic effect of tandem lesions, diffuse atherosclerosis, and microvascular dysfunction across the entire coronary tree, thereby providing a more comprehensive assessment of global myocardial flow reserve. A CT-FFR value ≤0.80 was considered positive for hemodynamically significant ischemia.

(3) Calcium Scoring and Stenosis Grading (17): Using the vendor-provided vessel analysis software, non-contrast coronary data were processed to automatically identify and quantify calcific lesions across the coronary tree, generating a total CACS. Concurrently, an experienced physician visually assessed the most severe stenosis in each major vessel on CCTA images and graded it according to CAD-RADS criteria: 0 (no stenosis), 1 (1%–24%, minimal), 2 (25%–49%, mild), 3 (50%–69%, moderate), 4 (70%–99%, severe), 5 (100%, occluded). The highest CAD-RADS grade per patient was recorded.

(4) Radiomic Feature Extraction and Model Construction: CCTA images from all 175 patients were anonymized and uploaded to the United Imaging Intelligent Research Platform (uAI Research Portal, version 2.0). The radiomics workflow was designed in accordance with the Image Biomarker Standardization Initiative (IBSI) guidelines (18). Image preprocessing included the following steps: all volumes were resampled to an isotropic voxel size of 1 × 1 × 1 mm^3^ using B-spline interpolation. Intensity normalization was applied by scaling voxel intensities to the range 0–2,000 HU, and outliers exceeding μ ± 3*σ* of the volume intensity distribution were clipped. For gray-level discretization, a fixed bin width of 25 HU was employed. To mitigate potential scanner-related batch effects between the uCT 860 and SOMATOM Force acquisitions, ComBat harmonization was applied to the extracted feature matrix, with scanner type as the batch variable.

For each coronary artery (LAD, LCX, RCA), the peri-coronary adipose tissue was automatically segmented as a three-dimensional volume of interest (VOI) defined as the tubular region extending radially 4 mm outward from the outer vessel wall, applying the same spatial definitions used for FAI measurement. Segmentation reproducibility was assessed in a random subset of 30 patients: two independent radiologists (with 8 and 6 years of cardiac CT experience, respectively) repeated the VOI segmentation, and the inter-observer agreement was evaluated using the intraclass correlation coefficient (ICC) for the final radiomics score. The ICC was 0.87 (95% CI 0.79–0.92), indicating good reproducibility. Intra-observer reproducibility was evaluated by having one radiologist repeat the segmentation after a 4-week interval; the intra-observer ICC was 0.91 (95% CI 0.85–0.95). To further assess feature stability, the ICC for each individual radiomic feature was calculated across the repeated segmentations; only features with an ICC ≥0.75 were retained for subsequent analysis. After this filtering step, 1,847 of the initial 2,264 features per vessel demonstrated acceptable stability.

From each VOI, a total of 2,264 radiomic features were initially extracted, comprising: 14 first-order histogram features, 8 three-dimensional shape features, and 2,242 higher-order texture features (Gray Level Co-occurrence Matrix, Gray Level Run Length Matrix, Gray Level Size Zone Matrix, Neighboring Gray Tone Difference Matrix). Features were computed on the original images and after application of 15 filters (wavelet decompositions, Laplacian of Gaussian with sigma values of 2.0, 3.0, and 5.0, and exponential, square, square root, logarithm, and gradient filters).

The entire dataset was randomly partitioned into a training set (80%, *n* = 140) and an internal hold-out test set (20%, *n* = 35) using stratified sampling to preserve the proportion of high-risk patients. To avoid information leakage, all feature selection and model tuning steps were performed exclusively within the training set. Within the training set, a nested cross-validation framework was adopted: the training set was further subdivided into 5 folds. In the inner loop, the maximum relevance minimum redundancy (mRMR) algorithm was applied to select the top 30 features with highest relevance to the high-risk label and minimal mutual redundancy; then least absolute shrinkage and selection operator (LASSO) logistic regression was performed with the optimal regularization parameter *λ* chosen via 10-fold cross-validation using the 1-standard-error rule. This inner procedure was repeated for each of the 5 outer training folds, yielding a robust set of non-zero coefficient features. The features that consistently appeared across at least 4 of the 5 folds were retained for the final model. The radiomics score was computed as the linear combination of the retained features weighted by their average coefficients across folds. The final coefficients for the RCA-PCAT radiomics model are provided in Supplementary Table S1. The linear predictor is given by: Radiomics score = *β*₀ + *β*₁ × original_firstorder_Mean + *β*₂ × original_glcm_Correlation+. +*β*_10_ × exponential_glszm_GrayLevelNonUniformity, where the intercept *β*₀ = −0.873 and the *β* coefficients are those listed in Supplementary Table S1. The model was then applied without modification to the fixed hold-out test set to obtain unbiased performance estimates.

(5) Combined Diagnostic Model Establishment: By comparing the diagnostic performance (primarily based on AUC) of the three vessel-specific radiomics models in the validation set, the optimal model and its corresponding vessel were selected. Subsequently, the predicted probability value (as a continuous variable) from this optimal radiomics model, along with CACS (continuous variable), the FAI value for that vessel (continuous variable), the minimum CT-FFR value for that vessel (dichotomous variable, ≤0.80 vs. >0.80), and the patient's highest CAD-RADS grade (ordinal categorical variable), were incorporated into a multivariable logistic regression model to construct a comprehensive combined diagnostic model.

### 2.5 Statistical analysis

All statistical analyses were performed using SPSS software (version 20.0) and the R environment (version 4.0.3). Continuous variables were first assessed for normality using the Kolmogorov–Smirnov test. Normally distributed data are presented as mean ± standard deviation and compared between groups using the independent samples *t*-test. Non-normally distributed data are presented as median (25th percentile, 75th percentile) and compared using the Mann–Whitney *U*-test. Categorical variables are presented as frequency (percentage) and compared using the chi-square test or Fisher's exact test (when expected cell counts were <5).

Continuous variables (FAI values, CACS, CT-FFR) were compared between the high-risk and low-risk groups using the Mann-Whitney *U*-test and presented as median (interquartile range). CAD-RADS grade was analyzed as an ordinal variable using the Mann–Whitney *U*-test; in addition, the proportion of patients with CAD-RADS >2 vs. ≤2 was compared using the chi-square test. Categorical variables were compared with the chi-square test or Fisher's exact test. For completeness, an additional analysis using dichotomized variables (median or clinical cut-offs) was performed and is reported in Supplementary Table S2; however, the primary inferences are drawn from the continuous-variable analyses.

Diagnostic performance was evaluated by plotting receiver operating characteristic (ROC) curves and calculating the area under the curve (AUC) with its 95% confidence interval (CI). This analysis was performed for the single radiomics score, CACS, FAI, CT-FFR, CAD-RADS grade, and the predicted probability from the final combined model. Comparison of AUCs was performed using DeLong's test. For the pairwise comparisons among the three vessel-specific radiomics models, *p*-values were adjusted using the Holm–Bonferroni correction to account for multiple testing. Variable importance in the logistic regression model was assessed via standardized regression coefficients or odds ratios with 95% CIs. To evaluate the clinical utility and calibration of the final combined model, decision curve analysis (DCA) was performed by plotting the net benefit against a range of threshold probabilities for high-risk status. Model calibration was assessed using the Hosmer–Lemeshow goodness-of-fit test, the calibration-in-the-large, and the calibration slope from a logistic calibration model. Overall predictive performance was quantified using the Brier score, with lower values indicating greater agreement between predicted probabilities and observed outcomes. Using the optimal cutoff determined by maximizing Youden's index in the training set, the model's diagnostic performance metrics—including sensitivity, specificity, positive predictive value (PPV), negative predictive value (NPV), and accuracy—were calculated in the test cohort. The key distinction is that AUC, calibration measures, the Brier score, and DCA evaluate the model itself, whereas sensitivity, specificity, PPV, NPV, and accuracy evaluate model performance at a specific cutoff threshold. DCA was conducted using the “rmda” (Risk Model Decision Analysis) package, and calibration analyses were conducted using the “rms” (Regression Modeling Strategies) package in R. All hypothesis tests were two-sided, and a *P*-value < 0.05 was considered statistically significant.

## 3 Results

### 3.1 Clinical characteristics

The age of all patients ranged from 36 to 80 years, with a median of 61 years (interquartile range [IQR]: 16). Body mass index (BMI) ranged from 18.2 to 35.0 kg/m², with a mean of 24.87 ± 3.12 kg/m^2^. Among the cohort, 134 patients had hypertension, 97 had hyperlipidemia, 43 had diabetes mellitus, 34 had a smoking history, and 37 had a family history of cardiovascular disease. There were 103 males and 72 females. According to the RF-CL model recommended by the 2024 ESC guidelines for CCS management, 98 patients were classified into the low-risk group (≤15%) and 77 into the high-risk group (>15%).

1. Statistical Results of CCTA-derived Imaging Metrics (Table 1)

**Table 1:** Comparison of continuous CCTA-derived imaging metrics between high-risk and low-risk CCS groups according to the RF-CL model.

CCTA-derived Imaging Metric	High-Risk CCS Group (RF-CL) (*n* = 77)	Low-Risk CCS Group (RF-CL) (*n* = 98)	*p*-value (Mann–Whitney U)
LCX-FAI (HU)	−85.10 (−96.28 to −74.90)	−86.43 (−95.65 to −76.80)	0.081
LAD-FAI (HU)	−80.20 (−88.55 to −71.30)	−84.65 (−91.40 to −76.90)	0.006
RCA-FAI (HU)	−72.15 (−82.00 to −63.40)	−78.50 (−86.75 to −69.10)	0.004
Plaque-distal CT-FFR	0.87 (0.79–0.94)	0.95 (0.91–0.98)	<0.001
Distal vessel CT-FFR	0.78 (0.73–0.82)	0.83 (0.80–0.88)	<0.001
CACS (Agatston units)	112.3 (21.4–386.5)	18.7 (0–95.6)	0.005
CAD-RADS grade^a^	3 (2–3)	2 (1–3)	0.039

a Values are median (interquartile range) unless otherwise indicated. CAD-RADS grade is ordinal and was compared using the Mann–Whitney *U*-test; the *p*-value in the table refers to this test. The chi-square test for the binary categorization CAD-RADS >2 vs. ≤2 is reported in Supplementary Table S2.

**Figure 1:**
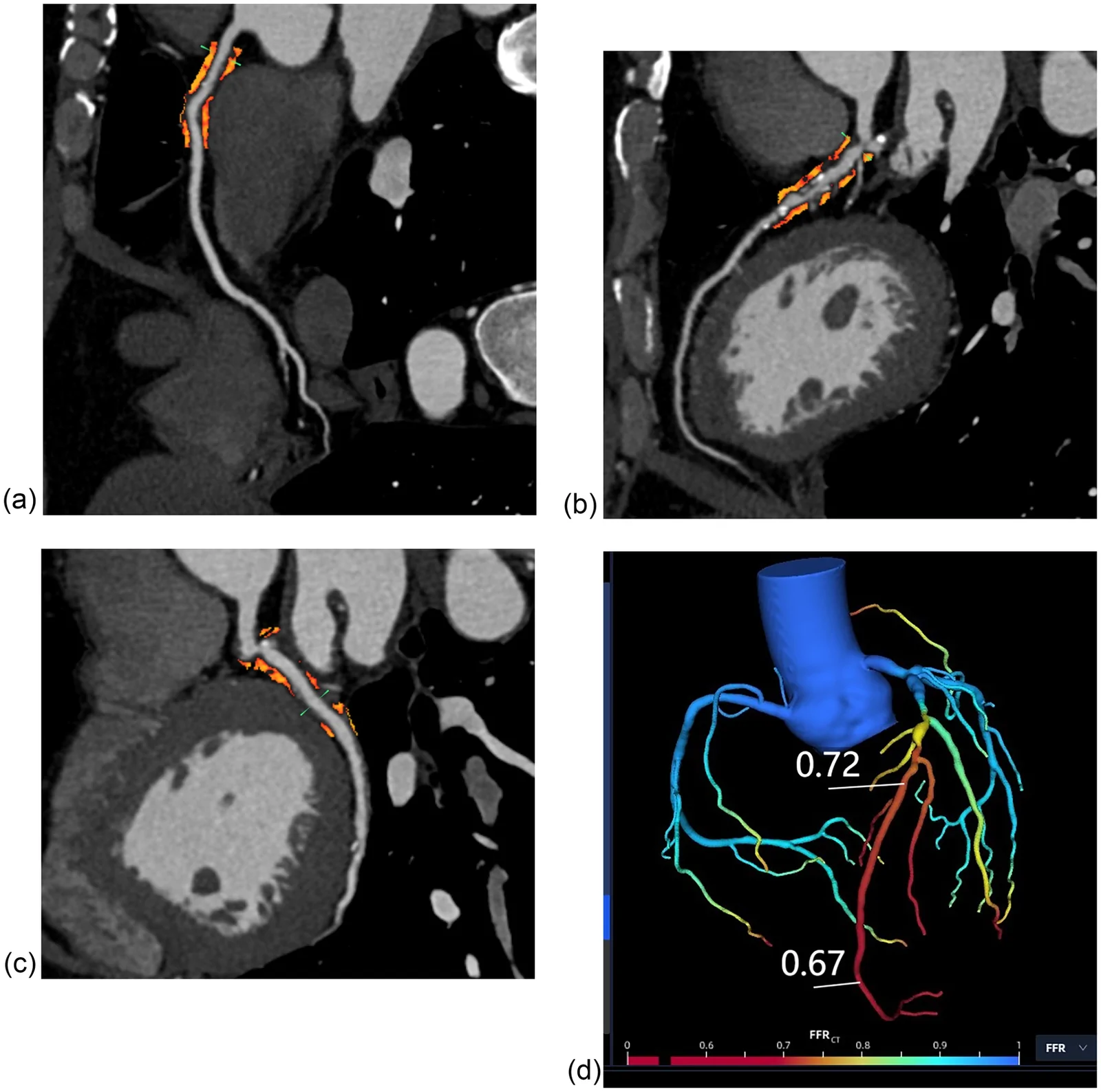
Representative imaging findings from a 48-year-old male patient classified as high risk by the RF-CL model. **(a)** RCA-FAI measurement (−79.90 HU) with the peri-coronary adipose tissue shaded in color; **(b)** LAD-FAI (−91.20 HU); **(c)** LCX-FAI (−83.03 HU); **(d)** CT-FFR color-coded map showing plaque-distal CT-FFR of 0.72 and distal vessel CT-FFR of 0.67. CACS was 476.5 and CAD-RADS grade 3. FAI, fat attenuation index; RCA, right coronary artery; LAD, left anterior descending; LCX, left circumflex; CT-FFR, computed tomography fractional flow reserve; CACS, coronary artery calcium score.

FAI values were not normally distributed; therefore, they are presented as median (interquartile range [IQR]) and were compared using the Mann–Whitney *U*-test. LCX-FAI ranged from −103.66 to −60.62 HU, with a median of −85.87 HU (IQR: 15.09 HU). LAD-FAI ranged from −100.91 to −64.47 HU, with a median of −82.49 HU. RCA-FAI ranged from −96.19 to −53.69 HU, with a median of −75.52 HU. CT-FFR measured distal to the most severe plaque ranged from 0.62 to 1.00, with a median of 0.92 (IQR: 0.10). Distal vessel CT-FFR ranged from 0.54 to 0.94, with a median of 0.81 (IQR: 0.09). CAD-RADS grading ranged from 0 to 4, with a median of 2 (IQR: 1). CACS ranged from 0 to 1,535.2, with a median of 51.1 (IQR: 186.0). Significant differences were observed between high- and low-risk CCS patients based on RF-CL for RCA-FAI (*p* = 0.004) and LAD-FAI (*p* = 0.006), as shown in Figures 1a–c, and for CACS (*p* = 0.005), CAD-RADS grade (Mann–Whitney *U*-test, *p* = 0.039; Table 1), plaque-distal CT-FFR (*p* < 0.001), and distal vessel CT-FFR (*p* < 0.001), with the CT-FFR findings shown in Figure 1d. The chi-square test for CAD-RADS >2 vs. ≤2 is reported in Supplementary Table S2. No significant difference was found for LCX-FAI (*p* = 0.081).

### 3.2 Diagnostic performance of individual imaging metrics and their combination with radiomics for identifying high-risk CCS patients based on RF-CL: ROC curve analysis

The performance of RCA-FAI (AUC = 0.620) was superior to that of LAD-FAI (AUC = 0.558) and LCX-FAI (AUC = 0.579). The AUC for CACS was 0.629, for CAD-RADS grade was 0.586, and for distal vessel CT-FFR (AUC = 0.782) was superior to plaque-distal CT-FFR (AUC = 0.698) (Table 2).

**Table 2:** Area under the ROC curve for individual imaging metrics in diagnosing high-risk CCS patients based on the RF-CL model.

CCTA-derived Imaging Metric	Area Under the ROC Curve (AUC)	95% Confidence Interval
LCX-FAI	0.579	0.492–0.666
LAD-FAI	0.558	0.472–0.644
RCA-FAI	0.62	0.535–0.704
Plaque-distal CT-FFR	0.698	0.617–0.780
Distal vessel CT-FFR	0.782	0.710–0.854
CAD-RADS Grade	0.586	0.501–0.671
CACS	0.629	0.545–0.714

**Figure 2:**
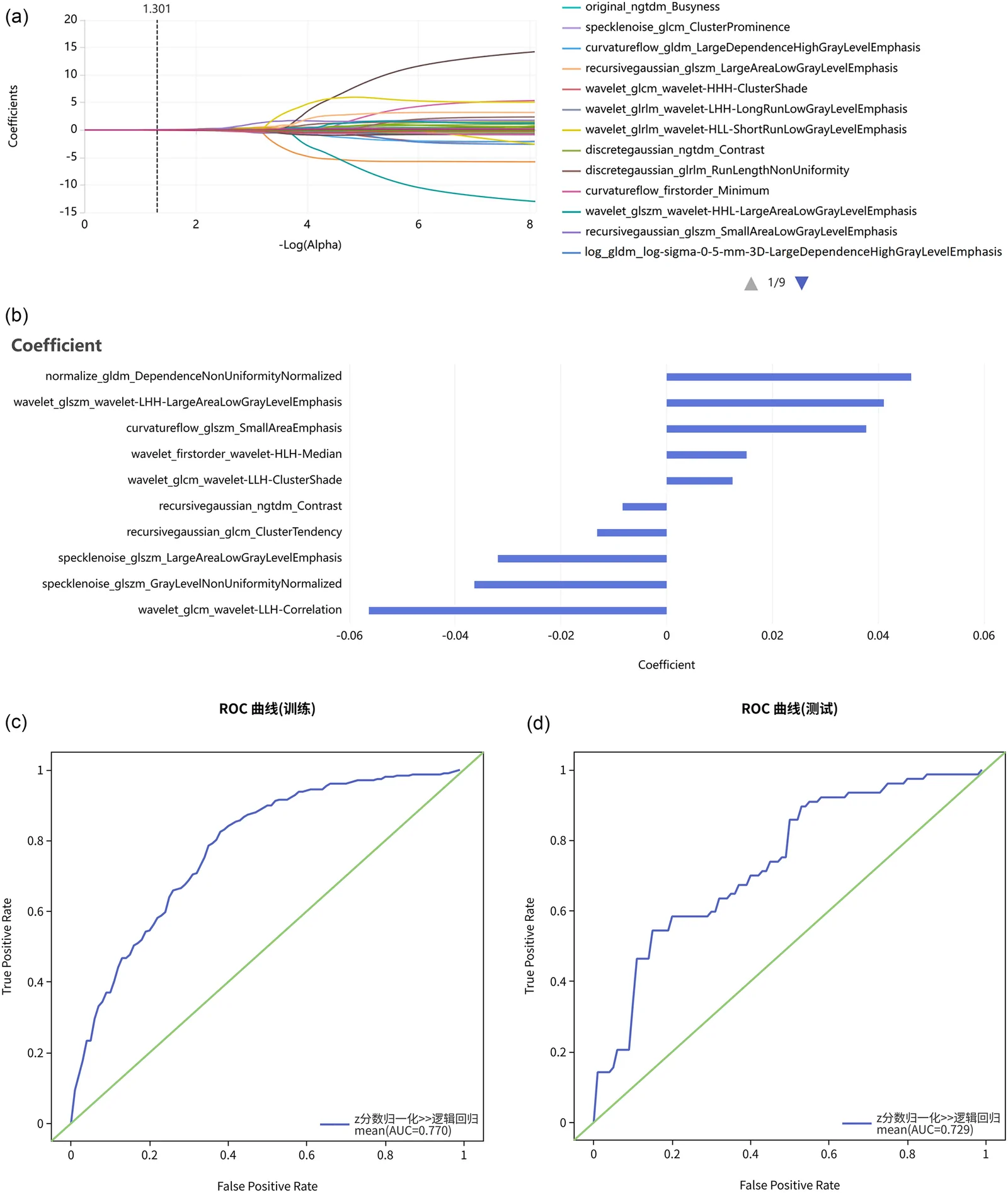
RCA-PCAT radiomics model feature selection and performance. **(a)** Heatmap of the top 10 selected radiomic features in the training set; rows represent patients, columns represent z-score-normalized features. **(b)** LASSO coefficient profile plot with the optimal *λ* (1-SE rule) indicated by the vertical dashed line. **(c)** Receiver operating characteristic (ROC) curve in the training set (AUC = 0.770, 95% CI 0.705–0.857). **(d)** ROC curve in the test set (AUC = 0.729, 95% CI 0.572–0.901). Shaded areas represent 95% confidence intervals. AUC, area under the curve.

**Figure 3:**
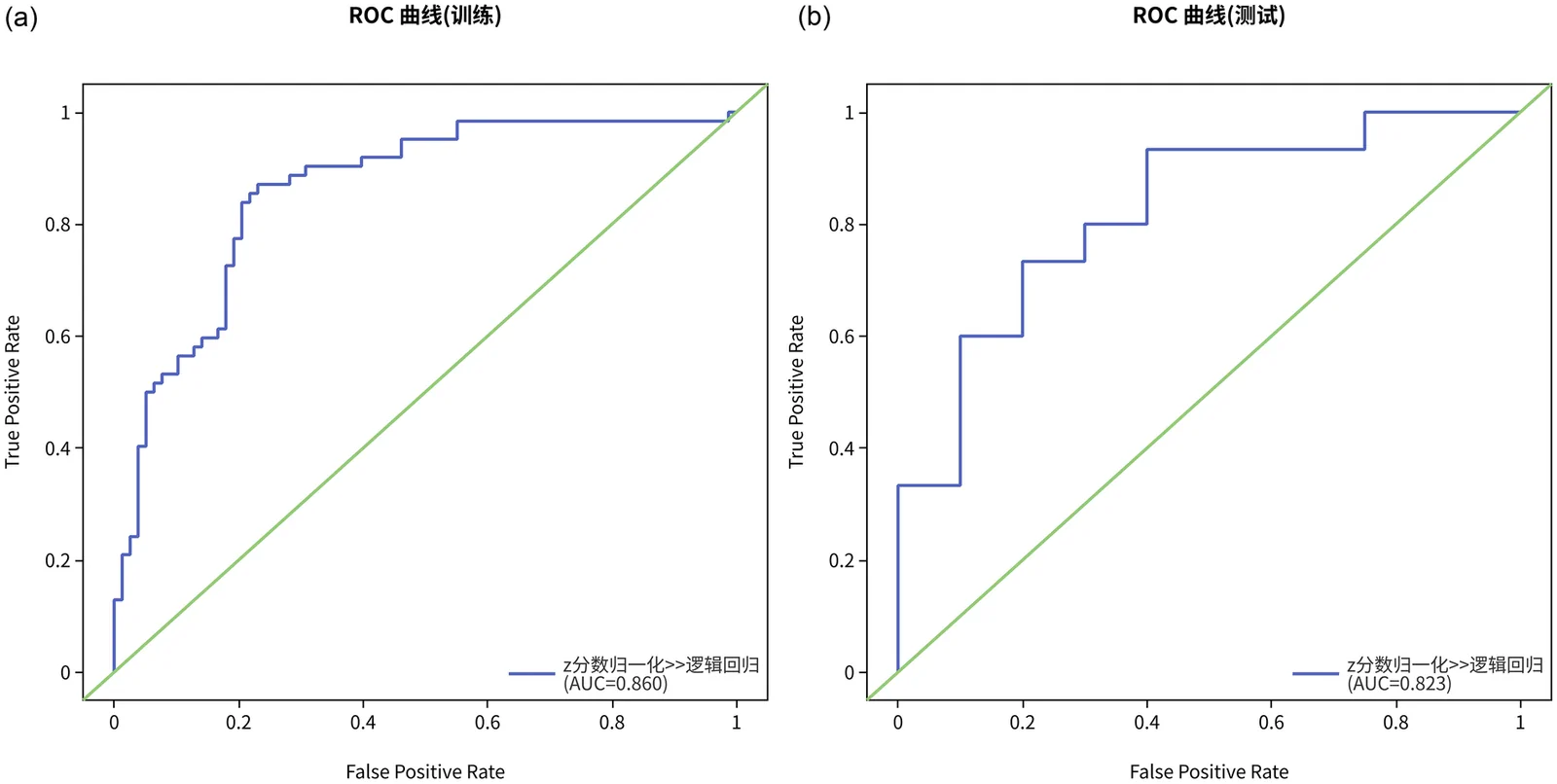
ROC curves for the multi-parameter combined model (RCA-PCAT radiomics + RCA-FAI + CACS + CAD-RADS grade + distal vessel CT-FFR). **(a)** Training set AUC = 0.860 (95% CI 0.798–0.922). **(b)** Test set AUC = 0.823 (95% CI 0.682–0.964).

Radiomics research was conducted using the United Imaging Intelligent Research Platform (uAI Research Portal). A total of 2,264 radiomic feature dimensions were extracted from the PCAT of each of the three coronary arteries. Following feature reduction, the ten optimal radiomic features were identified (Figures 2a,b). The results demonstrated that the diagnostic performance of the RCA-PCAT radiomics model was optimal (training set AUC = 0.770, test set AUC = 0.729) (Figures 2c,d), outperforming the LAD-PCAT (training set AUC = 0.762, test set AUC = 0.703) and LCX-PCAT (training set AUC = 0.747, test set AUC = 0.671) models (Table 3). The multi-parameter combined model integrating RCA-PCAT radiomics, RCA-FAI, CACS, CAD-RADS grade, and distal vessel CT-FFR demonstrated the best performance (training set AUC = 0.860, 95% CI 0.798–0.922; test set AUC = 0.823, 95% CI 0.682–0.964) (Figures 3a,b). The wide confidence interval in the test set reflects the limited sample size and should be interpreted with appropriate caution. Calibration of the combined model was excellent, with a calibration slope of 0.93 (95% CI 0.78–1.08), calibration-in-the-large of −0.04, and a Brier score of 0.16. The Hosmer–Lemeshow goodness-of-fit test yielded a *p*-value of 0.68, indicating no significant deviation from perfect calibration. Decision curve analysis showed that the combined model yielded a positive net benefit over the range of threshold probabilities from 10% to 60%, outperforming the treat-all and treat-none strategies as well as the RCA-PCAT radiomics-only model (Supplementary Figure S1). At the optimal threshold determined by maximizing Youden's index (predicted probability = 0.48), the combined model achieved a sensitivity of 78.4%, specificity of 80.6%, positive predictive value (PPV) of 76.5%, negative predictive value (NPV) of 82.3%, and accuracy of 79.5% in the test set.

**Table 3:** Area under the ROC curve for CCTA-based radiomics in diagnosing high-risk CCS patients based on the RF-CL model.

Radiomics Category	Training Set AUC (95% CI)	Test Set AUC (95% CI)
LCX-PCAT Radiomics	0.747 (0.678–0.837)	0.671 (0.501–0.857)
LAD-PCAT Radiomics	0.762 (0.696–0.851)	0.703 (0.540–0.882)
RCA-PCAT Radiomics	0.770 (0.705–0.857)	0.729 (0.572–0.901)
Combined Model: RCA-PCAT Radiomics + RCA-FAI + CACS + CAD-RADS Grade + Distal vessel CT-FFR	0.860 (0.798–0.922)	0.823 (0.682–0.964)

Combined Model: RCA-PCAT Radiomics + RCA-FAI + CACS + CAD-RADS Grade + Distal vessel CT-FFR 0.860 (0.798–0.922) 0.823 (0.682–0.964).

DeLong comparisons in the test set showed that the combined model had a significantly higher AUC than each individual predictor: vs. RCA-FAI (AUC 0.620, *p* = 0.012), vs. CACS (AUC 0.629, *p* = 0.021), vs. CAD-RADS (AUC 0.586, *p* = 0.008), vs. distal vessel CT-FFR (AUC 0.782, *p* = 0.048), and vs. RCA-PCAT radiomics alone (AUC 0.729, *p* = 0.039). Decision curve analysis showed that the combined model yielded a positive net benefit over the range of threshold probabilities from 10% to 60%, outperforming the strategy of considering all patients as high risk or none as high risk, as well as the RCA-PCAT radiomics-alone model (Supplementary Figure S1).

Multivariable logistic regression was performed with high-risk status as the dependent variable and the following continuous predictors: RCA-FAI (per 10 HU increase), CACS (per 100-unit increase), distal vessel CT-FFR (per 0.1-unit increase), CAD-RADS grade (per 1-category increase), and the RCA-PCAT radiomics score (per 1-unit increase). All predictors were entered simultaneously. The results were as follows: RCA-FAI, OR 1.32 (95% CI 1.08–1.61, *p* = 0.007); CACS, OR 1.18 (95% CI 1.04–1.34, *p* = 0.011); distal vessel CT-FFR, OR 0.42 (95% CI 0.26–0.68, *p* < 0.001); CAD-RADS, OR 1.45 (95% CI 1.10–1.92, *p* = 0.009); and RCA-PCAT radiomics score, OR 1.89 (95% CI 1.29–2.77, *p* = 0.001). This analysis confirms that each imaging variable contributes independently to the identification of RF-CL-defined high-risk status.

In the test set, DeLong pairwise comparisons among the three vessel-specific radiomics models did not reach statistical significance after Holm-Bonferroni correction (RCA vs. LAD, *p* = 0.61; RCA vs. LCX, *p* = 0.38; LAD vs. LCX, *p* = 0.67). Despite the overlapping confidence intervals, the RCA-PCAT model exhibited the highest point estimate of AUC and the most reproducible feature set across cross-validation folds. Based on these criteria, the RCA-PCAT radiomics score was chosen as the radiomic input for the combined model.

## 4 Discussion

Accurate risk stratification of chronic coronary syndrome (CCS) is crucial for guiding individualized therapy (19). This study aimed to systematically evaluate the diagnostic value of coronary computed tomography angiography (CCTA)-based pericoronary adipose tissue (PCAT) radiomic features, combined with multiple imaging parameters including the fat attenuation index (FAI), CT-derived fractional flow reserve (CT-FFR), coronary artery calcium score (CACS), and Coronary Artery Disease Reporting and Data System (CAD-RADS) grading, in identifying high-risk CCS patients as determined by a contemporary comprehensive risk model. The results demonstrated that RCA-FAI, CACS, CAD-RADS grade, and distal vessel CT-FFR were all significantly associated with high-risk status, whereas the discriminatory capacity of LCX-FAI was limited. Further analysis revealed that the radiomics model based on RCA-PCAT exhibited good discriminative performance, and the integrated model combining it with the aforementioned imaging metrics achieved the highest diagnostic accuracy. These findings suggest that the multi-parametric imaging information derived from a one-stop CCTA examination, particularly radiomics capturing deep texture features integrated with metrics reflecting hemodynamics and calcific burden, can provide a powerful supplement to risk assessment beyond traditional clinical risk models.

This study found that CCS patients have a favorable prognosis. However, both the latest 2024 European guidelines and China's first 2024 guidelines specifically for CCS patients recommend pre-test assessment of the likelihood of obstructive coronary artery disease (CAD) in CCS to guide referral for downstream diagnostic testing (20). Compared to the traditional clinical pre-test probability (PTP) model, which primarily includes three indicators—chest pain characteristics (typical angina, atypical angina, non-anginal chest pain), sex, and age—the latest RF-CL model recommended by the 2024 ESC guidelines improves the predictive ability for obstructive CAD and the discrimination of cardiovascular risk. It reclassifies patients who do not require further diagnostic testing into lower probability categories and increases the number of subjects with a very low probability (≤5%) of obstructive CAD (20). Furthermore, multiple studies based on Asian populations, including Chinese, have shown that CACS has a modifying effect on the predicted risk probability of the RF-CL model. Incorporating CACS into the RF-CL model can more accurately predict major adverse cardiovascular events (MACE). A low CACS can down-classify high-risk probability, avoiding unnecessary further testing, helping to optimize downstream clinical management, and potentially alleviating healthcare burden (21, 22).

The RF-CL model estimates the pre-test probability of obstructive CAD and the 10-year risk of cardiovascular death or non-fatal MI by incorporating clinical variables, including age, sex, symptom characteristics, and cardiovascular risk factors. CACS, when added to the RF-CL framework, acts as a potent modifier: a low CACS (commonly <100 or =0) can substantially downgrade the predicted risk probability, whereas a high CACS (>300) upgrades it, thereby reclassifying patients into more appropriate risk categories (21, 22). In our study, CACS was significantly higher in the high-risk RF-CL group. It is important to note that the RF-CL score was derived solely from clinical variables and did not include CACS or any CCTA-derived parameter. The observed association between high CACS and RF-CL-defined high-risk status therefore reflects the established link between calcific plaque burden and underlying cardiovascular risk profile, not circularity. This independence ensures that the imaging predictors in our combined model provide complementary information beyond the clinical risk classification. However, beyond this intrinsic relationship, CACS also provides complementary structural information that is not entirely captured by the clinical variables of the RF-CL model. When combined with FAI (inflammatory status), CT-FFR (functional significance), and PCAT radiomics (tissue heterogeneity), the multi-parametric model captures different pathophysiological axes—calcific burden, inflammation, ischemia, and perivascular tissue remodeling—thereby improving discrimination beyond the RF-CL model alone.

Our study also found a statistically significant difference in CACS between the high- and low-risk groups defined by RF-CL. ROC analysis indicated that CACS had relatively good performance in identifying high-risk patients (AUC = 0.629). This result may indirectly corroborate the influence of CACS on the predicted risk probability of the RF-CL model. When comparing high- and low-risk patients stratified by the comprehensive risk model, elevated FAI around the RCA and LAD was associated with high-risk status, while LCX-FAI showed no significant difference. This discrepancy may stem from anatomical variations in vessel course, lumen size, and the myocardial territory supplied by different coronary arteries, which influence the microenvironment and inflammatory response characteristics of their surrounding adipose tissue (23). The RCA and LAD, as major epicardial vessels, may have perivascular fat that is more sensitive to atherosclerotic inflammatory responses. In contrast, assessment of the LCX is susceptible to interference from cardiac motion, proximity to atrial structures, and uneven fat distribution, potentially compromising the robustness of its attenuation index as an inflammatory biomarker (24). Indeed, studies have shown that the LCX territory is more frequently affected by motion artifacts on CCTA, which can degrade FAI measurement reliability. In a sensitivity analysis excluding nine patients with severe motion artifacts in the LCX region, the difference in LCX-FAI between high- and low-risk groups remained non-significant, supporting the notion that LCX-FAI has limited discriminatory ability in this population. CACS and CAD-RADS grade were significantly higher in the high-risk group, consistent with their pathological basis reflecting long-term calcific burden and anatomical stenosis severity, respectively, confirming the fundamental role of structural disease in risk stratification. Distal vessel CT-FFR demonstrated better discriminatory ability than plaque-distal CT-FFR for identifying RF-CL-defined high-risk status. This may reflect the ability of the distal measurement to capture the summed effect of serial lesions, diffuse atherosclerosis, and microvascular impairment. However, this finding should be interpreted cautiously: our endpoint is RF-CL classification, not observed MACE. Whether distal vessel CT-FFR similarly outperforms plaque-distal CT-FFR for predicting hard outcomes requires confirmation in prospective studies with clinical event endpoints (23). Previous studies also support the core value of CT-FFR in assessing hemodynamically significant ischemia. Our study further clarifies its potential application in distinguishing patients across different clinical risk strata.

Research has found that bioactive molecules and adipokines produced during PCAT metabolism can modulate the inflammatory environment of the vessel wall and adjacent perivascular regions, activating endothelial cells and promoting leukocyte recruitment. PCAT-induced dysregulation of lipid metabolism and adipocyte-derived cytokines may indirectly affect the arterial wall by promoting oxidative stress and altering the function of vascular endothelial cells, smooth muscle cells, and macrophages. In summary, the metabolic crosstalk between PCAT and the arterial wall plays a key role in the development of atherosclerosis, promoting endothelial dysfunction, inflammatory responses, and smooth muscle cell proliferation, thereby influencing the occurrence and progression of atherosclerotic plaques (25, 26). Earlier studies found that lesion-specific pericoronary FAI elevation on CCTA was an independent factor influencing MACE in patients with type 2 diabetes. Combining lesion-specific pericoronary FAI with CACS provided incremental predictive ability for MACE in these patients (27). A multicenter study with a median follow-up of 2.7 years found that a higher FAI score significantly impacted the risk of cardiac death or MACE, and FAI in any coronary artery independently predicted cardiac death and MACE (28). However, van Rosendael et al., after a long-term follow-up with a median of 9.5 years in patients with suspected CAD, found that neither the average FAI of all coronary lesions nor the FAI of only the most stenotic lesion could predict MACE (29). This suggests that the predictive ability of FAI for future adverse events in CAD may be uncertain, and its value in predicting MACE requires further exploration, especially the controversial ability of LCX-FAI to assess cardiovascular risk warrants additional investigation. In our study, PCAT FAI showed no statistical difference between the high- and low-risk groups based on the RF-CL model for the LCX, but it did for the RCA and LAD. Furthermore, compared to the LAD and LCX, RCA-FAI also showed the relatively best AUC (0.620) for diagnosing high-risk patients. RCA-FAI appears to have a good effect on predicting MACE, and its predictive value for CAD prognosis is widely recognized.

PCAT is a subcompartment of the larger epicardial adipose tissue (EAT) depot, which directly contacts the coronary adventitia and shares the same microcirculatory environment. Beyond PCAT, global EAT—assessable by CT, magnetic resonance imaging, and echocardiography—provides complementary information on metabolic activity, inflammatory status, and cardiovascular risk. A recent comprehensive multimodal imaging review highlights that EAT quantification and tissue characterization, including radiomics analysis, can improve risk stratification in coronary artery disease beyond traditional risk factors. In light of this, our PCAT-focused radiomics model may capture a local inflammatory signal that is part of a broader EAT-mediated pathophysiological process. Future studies integrating both PCAT and total EAT radiomics could further enhance the precision of cardiovascular risk assessment.

Our study demonstrated that radiomic features from the PCAT of all three coronary arteries have the potential to identify the risk status of CCS patients based on the RF-CL model (both training and test set AUCs >0.6), with the RCA model performing best (training set AUC = 0.770, test set AUC = 0.729) and outperforming FAI. Recent studies have shown that CCTA-based PCAT radiomic features are superior to FAI models in discriminating unstable angina from stable angina, suggesting that integrated models combining radiomics with clinical features can further improve the diagnosis and differentiation of unstable angina (30). These findings consistently indicate that the diagnostic and predictive performance of PCAT radiomics is superior to FAI, aligning with our results. Ye et al. found that CCTA-based PCAT radiomics could effectively differentiate coronary segments with and without plaque, suggesting that machine learning-based radiomic features of perivascular adipose tissue can predict the inflammatory state around atherosclerotic plaques (31). Another study found significantly elevated levels of CD31 (platelet endothelial cell adhesion molecule-1, PECAM-1), MCP-1 (monocyte chemoattractant protein-1, CCL2), and leptin in PCAT based on gene expression analysis, and PCAT radiomic features showed strong correlations with the expression of CD31 and MCP-1. Serological markers such as CD31, ADP (adenosine diphosphate), and IL-6 (interleukin-6) were significantly correlated with PCAT texture features, while TNF-α (tumor necrosis factor-alpha) was correlated with first-order features. That study concluded that PCAT radiomic features in CAD patients effectively reflect the pathological state of PCAT and have potential as a novel imaging biomarker (32). Based on the literature and our findings, we posit that compared to the single-parameter density analysis of FAI, radiomics employs high-throughput image feature extraction technology combined with machine learning to perform multi-dimensional analysis of perivascular adipose tissue heterogeneity, generating high-order quantitative parameters that can better reveal biological information not captured by traditional imaging.

Chen et al. (14) found that lesion-specific PCAT radiomics models and clinical-radiomics models based on CCTA, determined by the lowest CT-FFR value, could effectively predict MACE and were significantly superior to clinical models based on traditional risk factors. In our study, plaque-distal CT-FFR showed a statistical difference between the high- and low-risk groups, consistent with the general pattern reported in the literature. Additionally, we found that distal vessel CT-FFR also differed significantly between the groups. Compared to plaque-distal CT-FFR, ROC analysis showed that distal vessel CT-FFR had better performance in identifying high-risk patients. Although literature often uses CT-FFR to predict MACE as an endpoint, whereas our study aimed solely at identifying high-risk patients, this phenomenon might suggest that CT-FFR values from different vascular regions have inconsistent predictive and discriminative abilities for high-risk CCS patients. Indeed, domestic literature reports on the application value of distal coronary CT-FFR in CAD are relatively scarce. In summary, our research group speculates that a stenosis-distal CT-FFR ≤0.8 may indicate a higher risk of future MACE, while a coronary distal vessel CT-FFR ≤0.8 might signify that the patient is already in a high-risk state for potentially developing obstructive CAD. Its value is equally worthy of attention and further exploration.

Diagnostic performance analysis revealed that among single imaging parameters, distal vessel CT-FFR had the highest discriminatory value, followed by CACS and RCA-FAI. This highlights the importance of functional ischemia assessment in immediate risk stratification, with its efficacy surpassing that of indicators solely reflecting inflammation or calcification (33). However, the radiomic features extracted from RCA-PCAT demonstrated diagnostic performance superior to that vessel's FAI and even comparable to distal vessel CT-FFR. This suggests that radiomics can capture complex information about tissue heterogeneity and spatial arrangement not encompassed by FAI, potentially providing a deeper characterization of the biological activity and pathological state of perivascular adipose tissue (34). Ultimately, the combined model integrating the RCA radiomics score, RCA-FAI, CACS, CAD-RADS grade, and distal vessel CT-FFR achieved the best diagnostic performance. This model amalgamates multi-dimensional information spanning morphology, function, and biological activity: CACS and CAD-RADS grade provide the “static” structural burden of disease, CT-FFR reflects “dynamic” flow limitation, while radiomics and FAI attempt to quantify the “active” inflammatory milieu of the vessel wall (35). This multi-parameter strategy demonstrates a proof-of-concept for integrating anatomic, functional, and inflammatory imaging markers within a unified framework. Whether this imaging-based identification of RF-CL high-risk patients translates into improved therapeutic decision-making or clinical outcomes remains to be established through prospective interventional studies (36).. Compared to previous literature focusing on predicting long-term hard endpoints, this study directly addresses the current clinical need for high-risk identification, demonstrating the practical value of multi-parametric imaging models in this scenario (36).

This study has several limitations. First, as a retrospective study, selection bias is inevitable. Second, the reference standard in this study was the RF-CL-defined high-risk status (risk >15%), not observed major adverse cardiovascular events (MACE), myocardial infarction, cardiac death, or hospitalization. This design choice implies that the developed model primarily identifies patients already classified as high risk by the RF-CL framework; its ability to predict actual clinical outcomes remains unproven. Therefore, conclusions should not be extrapolated to long-term prognostic prediction with hard endpoints without confirmatory outcome-based studies. Furthermore, the reproducibility of radiomic features is susceptible to variations in image acquisition parameters, reconstruction algorithms, and segmentation methods. Although this study was conducted under a fixed platform workflow to maintain internal consistency, the generalizability of the results across centers and devices requires further validation in prospective, large-sample, multi-center cohorts. Future research should explore the seamless integration of such multi-parametric imaging models into clinical workflows and evaluate whether their guidance of treatment decisions can ultimately improve patient outcomes. Third, radiomic feature extraction was performed exclusively with the United Imaging uAI Research Portal (version 2.0). Radiomics features are known to be dependent on the software platform, reconstruction algorithms, and acquisition parameters. Although ComBat harmonization was applied to mitigate between-scanner differences, the reproducibility of our feature set and model performance across alternative radiomics platforms and different CT systems has not been assessed. This limits the immediate transportability of the model and should be addressed in future multicenter studies. Fourth, all models were validated on an internal hold-out split from the same cohort; no independent external validation cohort was available. Consequently, the reported performance estimates may be optimistic and the generalizability of the radiomics features and the combined model to other centers, scanners, and populations remains to be established. A prospective, multicenter study with external validation is essential before clinical application can be considered. Although the combined imaging model accurately identifies patients already classified as high risk by RF-CL, the present study provides no evidence that this reclassification translates into improved patient management, treatment decisions, or clinical outcomes. Prospective trials are required to determine whether an imaging-augmented risk stratification strategy leads to changes in therapy and, ultimately, to a reduction in adverse events. Additionally, because the RF-CL risk score incorporates CACS and, in some of its component scores, may be influenced by the extent of atherosclerosis that is also correlated with functional stenosis severity, the associations of CACS and CT-FFR with the high-risk label are partly expected and may involve circularity. The observed discriminatory performance of these variables should therefore be interpreted as demonstrating their alignment with the RF-CL classification rather than as independent validation. Confirmatory studies with hard clinical endpoints are essential to disentangle these relationships.

In conclusion, this study demonstrates that CCTA-based radiomics, combined with FAI, CT-FFR, CACS, and CAD-RADS grading, can accurately identify CCS patients who are categorized as high-risk by the RF-CL model. While this multi-dimensional imaging approach shows promise for refining risk stratification in clinical practice, it must be emphasized that the current model identifies RF-CL-defined high-risk status and does not predict observed adverse events. Therefore, prospective multicenter studies with hard cardiovascular endpoints (e.g., MACE, cardiac death, myocardial infarction) are necessary to validate its true prognostic value and to assess whether imaging-guided risk reclassification translates into improved patient management and outcomes. Furthermore, the test-set AUC of the combined model showed a wide 95% confidence interval (0.682–0.964), indicating considerable uncertainty around the point estimate that is primarily attributable to the modest sample size. While the lower bound remains above the null value of 0.70, the results must be interpreted with caution. Rigorous external validation in larger, independent cohorts is required to confirm the stability and generalizability of these performance estimates.

## Data Availability

The datasets presented in this article are not readily available because Data are available from the corresponding author upon reasonable request and with appropriate permissions. Requests to access the datasets should be directed to Zhang Yu; zhangyu105fsk@163.com.
