# Supplementary material for: Preliminary exploration of the value of pericoronary adipose tissue radiomics in identifying high-risk patients with chronic coronary syndrome: a retrospective Chinese cohort study
Source: Front Med (Lausanne). 2026 Sep 3;13:1797905. doi: 10.3389/fmed.2026.1797905 (PMC13581928; doi:10.3389/fmed.2026.1797905)
Supplement: Supplementary file 1 [file Table1.docx]

Supplementary Table 1. Final coefficients of the RCA‑PCAT radiomics model

| **Feature name** | **Coefficient** |
| --- | --- |
| original_firstorder_Mean | 0.423 |
| original_glcm_Correlation | −0.312 |
| wavelet-LHL_firstorder_Skewness | 0.287 |
| wavelet-HHH_glrlm_RunEntropy | −0.256 |
| log-sigma-3-0-mm-3D_glszm_ZoneEntropy | 0.198 |
| wavelet-LLH_ngtdm_Contrast | −0.174 |
| original_glrlm_LongRunEmphasis | 0.151 |
| wavelet-HLL_firstorder_Kurtosis | −0.133 |
| square_glcm_InverseVariance | 0.119 |
| exponential_glszm_GrayLevelNonUniformity | −0.097 |
| Intercept | −0.873 |

Abbreviations: RCA, right coronary artery; PCAT, pericoronary adipose tissue. Coefficients correspond to the linear predictor of the logistic regression model after LASSO regularization.
