# Supplementary material for: Preliminary exploration of the value of pericoronary adipose tissue radiomics in identifying high-risk patients with chronic coronary syndrome: a retrospective Chinese cohort study
Source: Front Med (Lausanne). 2026 Sep 3;13:1797905. doi: 10.3389/fmed.2026.1797905 (PMC13581928; doi:10.3389/fmed.2026.1797905)
Supplement: Supplementary file 2 [file Table2.docx]

Supplementary Table 2. Chi‑square test results of dichotomized imaging metrics between high‑ and low‑risk CCS groups (supplementary to primary continuous‑variable analyses)

| **CCTA‑derived imaging metric** | **High‑risk CCS group (RF‑CL) (n=77)** | **Low‑risk CCS group (RF‑CL) (n=98)** | **p‑value** |
| --- | --- | --- | --- |
| LCX‑FAI |  |  | 0.081 |
| ≤ −85.87 HU (median) | 33 (18.9%) | 55 (31.4%) |  |
| > −85.87 HU | 44 (25.1%) | 43 (24.6%) |  |
| LAD‑FAI |  |  | 0.006 |
| ≤ −82.49 HU (median) | 28 (16.0%) | 56 (32.0%) |  |
| > −82.49 HU | 49 (28.0%) | 42 (24.0%) |  |
| RCA‑FAI |  |  | 0.004 |
| ≤ −75.52 HU (median) | 31 (17.7%) | 61 (34.9%) |  |
| > −75.52 HU | 46 (26.3%) | 37 (21.1%) |  |
| Plaque‑distal CT‑FFR |  |  | <0.001 |
| ≤ 0.80 (clinical cut‑off) | 19 (10.9%) | 3 (1.7%) |  |
| > 0.80 | 58 (33.1%) | 95 (54.3%) |  |
| Distal vessel CT‑FFR |  |  | <0.001 |
| ≤ 0.80 (clinical cut‑off) | 63 (36.0%) | 21 (12.0%) |  |
| > 0.80 | 14 (8.0%) | 77 (44.0%) |  |
| CAD‑RADS grade |  |  | 0.039 |
| ≤ 2 | 33 (18.9%) | 55 (31.4%) |  |
| > 2 | 44 (25.1%) | 43 (24.6%) |  |
| CACS |  |  | 0.005 |
| ≤ 51.1 (median) | 29 (16.6%) | 59 (33.7%) |  |
| > 51.1 | 48 (27.4%) | 39 (22.3%) |  |

Data are presented as frequency (percentage). Continuous variables were dichotomized at the overall median or at the clinically accepted cut‑off of 0.80 for CT‑FFR. p‑values were calculated using the chi‑square test.

CCS, chronic coronary syndrome; CCTA, coronary computed tomography angiography; FAI, fat attenuation index; LAD, left anterior descending; LCX, left circumflex; RCA, right coronary artery; CT‑FFR, computed tomography fractional flow reserve; CAD‑RADS, Coronary Artery Disease Reporting and Data System; CACS, coronary artery calcium score.
